# PARK7 promotes repair in early steroid-induced osteonecrosis of the femoral head by enhancing resistance to stress-induced apoptosis in bone marrow mesenchymal stem cells via regulation of the Nrf2 signaling pathway

**DOI:** 10.1038/s41419-021-04226-1

**Published:** 2021-10-13

**Authors:** Fei Zhang, Yanglin Yan, Wuxun Peng, Lei Wang, Tao Wang, Zhihong Xie, Hong Luo, Jian Zhang, Wentao Dong

**Affiliations:** 1 Department of Orthopedics, The Affliated Hospital of Guizhou Medical University, Guiyang, Guizhou 550004 China; 2 School of Clinical Medicine, Guizhou Medical University, Guiyang, Guizhou 550004 China

**Keywords:** Diseases, Stem-cell research

## Abstract

Novel therapies for the treatment of early steroid-induced osteonecrosis of the femoral head (SONFH) are urgently needed in orthopedics. Transplantation of bone marrow mesenchymal stem cells (BMSCs) provides new strategies for treating this condition at the early stage. However, stress-induced apoptosis of BMSCs transplanted into the femoral head necrotic area limits the efficacy of BMSC transplantation. Inhibiting BMSC apoptosis is key to improving the efficacy of this procedure. In our previous studies, we confirmed that Parkinson disease protein 7 (PARK7) is active in antioxidant defense and can clear reactive oxygen species (ROS), protect the mitochondria, and impart resistance to stress-induced apoptosis in BMSCs. In this study, we investigated the mechanism driving this PARK7-mediated resistance to apoptosis in BMSCs. Our results indicate that PARK7 promoted the disintegration of nuclear factor (erythroid-derived 2)–like 2 (Nrf2)/Kelch-like echinacoside–associated protein 1 (Keap1) complex. The free Nrf2 then entered the nucleus and activated the genetic expression of manganese superoxide dismutase (*MnSOD*), catalase (*CAT*), glutathione peroxidase (*GPx*), and other antioxidant enzymes that clear excessive ROS, thereby protecting BMSCs from stress-induced apoptosis. To further explore whether PARK7-mediated resistance to stress-induced apoptosis could improve the efficacy of BMSC transplantation in early-stage SONFH, we transplanted BMSCs-overexpressing PARK7 into rats with early-stage SONFH. We then evaluated the survival of transplanted BMSCs and bone regeneration in the femoral head necrotic area of these rats. The results indicated that PARK7 promoted the survival of BMSCs in the osteonecrotic area and improved the transplantation efficacy of BMSCs on early-stage SONFH. This study provides new ideas and methods for resisting the stress-induced apoptosis of BMSCs and improving the transplantation effect of BMSCs on early-stage SONFH.

## Introduction

Steroid-induced osteonecrosis of the femoral head (SONFH), a joint dysfunction caused by long-term heavy use of glucocorticoids, results in a high rate of disability. Effective early-stage treatment strategies are urgently needed in the field of orthopedics [1, 2]. In recent years, cells with bone regeneration ability such as bone marrow mesenchymal stem cells (BMSCs) have been used to treat early-stage SONFH. However, the survival of the cells seeded into the osteonecrotic area is key to achieving successful transplantation [3, 4]. In our previous study, we used BMSCs to construct tissue-engineered bone for the repair of early-stage SONFH. However, transplantation efficacy was not satisfactory mainly because numerous BMSCs transplanted into the osteonecrotic area underwent stress-induced apoptosis [5].

Oxidative stress (OS) plays an important role in SONFH development and in osteonecrosis repair [6, 7]. OS results from increased levels of reactive oxygen species (ROS), decreased ROS-scavenging ability, or imbalance in oxidation and antioxidant defense system, leading to excessive ROS production in cells. These events can alter the cellular redox state; damage deoxyribonucleic acid (DNA), proteins, lipids, and other biological macromolecules; change cellular structure and function; and trigger a variety of apoptotic signaling pathways, such as those involving tumor protein 53 (p53) and p38/mitogen-activated protein kinase (p38/MAPK), to induce apoptosis [8–12]. An OS microenvironment exists in the osteonecrotic area of the femoral head; therefore, many BMSCs transplanted into this area undergo stress-induced apoptosis, which inhibits the reparative effectiveness of this therapy in early-stage SONFH [13, 14]. Therefore, enhancing resistance to OS in BMSCs will likely improve their therapeutic efficacy in early-stage SONFH.

The highly conserved Parkinson disease protein 7 (PARK7) exists as a homologous dimer composed of 189 amino acids. PARK7 is widely expressed in various cells and tissues, including BMSCs. As an active oxygen scavenger and antioxidant, PARK7 has an important role in cell survival and resistance to OS [15–18]. The results obtained in our previous study confirmed that PARK7 enhances OS resistance in BMSCs, thereby enabling them to resist stress-induced apoptosis [19]. However, the molecular mechanism of PARK7-mediated resistance to OS, and whether PARK7-mediated resistance to stress-induced apoptosis improves the efficacy of BMSC transplantation in early-stage SONFH, remain unclear.

In this study, we further explored the molecular mechanism underlying PARK7-mediated resistance to OS and evaluated the role of PARK7 in BMSC transplantation for the repair of early-stage SONFH. Our study will provide new methods for promoting resistance to stress-induced apoptosis in BMSCs and improving their therapeutic effectiveness in early-stage SONFH.

## Results

### PARK7 enhances BMSC resistance to OS

**Fig. 1:**
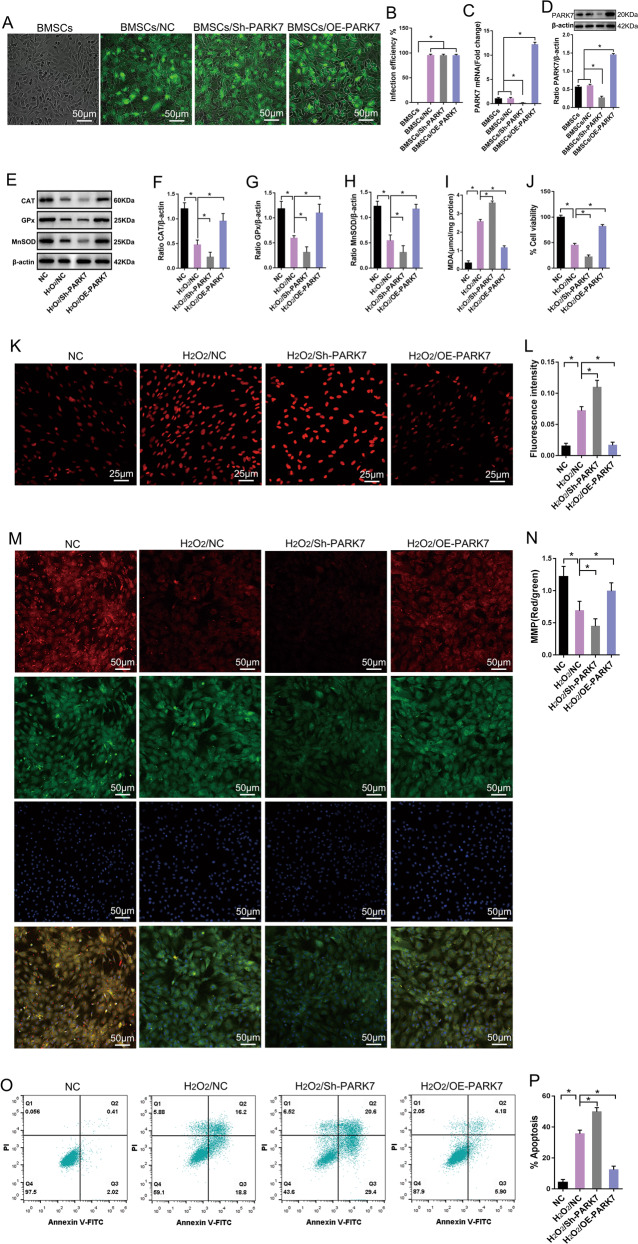
PARK7 enhances the ability of BMSCs to resist oxidative stress. After BMSCs were transfected with the PARK7 overexpression or PARK7-interfering lentivirus: **A** Expression levels of the reporter gene *GFP* were observed using an inverted fluorescence microscope (*n* = 6). *PARK7* Parkinson disease protein 7, *BMSCs* bone marrow mesenchymal stem cells, *GFP* green fluorescent protein, *NC* negative control, *Sh-PARK7* short-hairpin ribonucleic acid of *PARK7*, *OE-PARK7* overexpression of PARK7. **B** GFP expression levels were quantified in BMSCs as shown in **A** (*n* = 6). **C** RT-qPCR analysis of PARK7 mRNA expression in BMSCs (*n* = 3). RT-qPCR, real-time quantitative polymerase chain reaction. **D** Immunoblot analysis of PARK7 expression levels in BMSCs (*n* = 3). After PARK7 expression was upregulated or downregulated in BMSCs, BMSCs were treated with H_2_O_2_ to simulate oxidative stress. **E** Immunoblot analysis of CAT, GPx, and MnSOD expression in BMSCs (*n* = 3). *MnSOD* manganese superoxide dismutase, *CAT* catalase, *GPx* glutathione peroxidase, *H*_*2*_*O*_*2*_ hydrogen peroxide. **F** Quantification of CAT expression is shown in **E** (*n* = 3). **G** Quantification of GPx expression is shown in **E** (*n* = 3). **H** Quantification of MnSOD expression is shown in **E** (*n* = 3). **I** Detection of MDA levels using thiobarbituric acid assay (*n* = 6). *MDA* malondialdehyde. **J** Detection of cell viability using Cell Counting Kit-8 (*n* = 6). **K** Detection of ROS levels using DHE (*n* = 6). *ROS* reactive oxygen species, *DHE* dihydroethidium. **L** Quantification of ROS levels is shown in **K** (*n* = 6). **M** Detection of MMP using a JC-1 assay (*n* = 5). *MMP* mitochondrial membrane potential, *DAPI* 4′,6-diamidino-2-phenylindole; JC-1, 5,5′,6,6′-tetrachloro-1,1′,3,3′-tetraethyl- imidacarbocyanine. **N** Quantification of MMP is shown in **M** (*n* = 5). **O** Flow cytometry analysis of apoptosis (*n* = 5). *PI* propidium iodide, *FITC* fluorescein isothiocyanate. **P** Quantification of apoptosis levels is shown in **O** (*n* = 6). All data are represented as mean ± standard deviation (SD). *P < 0.05. Differences were tested using one-way analysis of variance (ANOVA) with Tukey’s post hoc test (**B**–**D**, **F**–**J**, **L**, **N**, **P**).

First, we explored the role of PARK7 in BMSC response to OS. For this, we inserted the coding sequence region of the *PARK7* gene into an overexpressed lentiviral vector (OE-PARK7). We then designed a short-hairpin ribonucleic acid-targeting *PARK7* and inserted it into an interfering lentiviral vector (Sh-PARK7). After we successfully isolated and cultured BMSCs (Fig. S1), these lentiviruses were consequently transfected into BMSCs. At 5 days after transfection, we observed that green fluorescent protein was successfully expressed in BMSCs, and the transfection efficiency was >90% (Fig. 1A, B). The results of real-time quantitative polymerase chain reaction (qPCR) and immunoblotting also confirmed that PARK7 expression was successfully up or downregulated in BMSCs (Fig. 1C, D). Finally, we treated BMSCs with a high concentration of hydrogen peroxide (H_2_O_2_; 1000 μM) for 24 h to simulate conditions of OS. Our results indicate that after prolonged exposure to OS, BMSCs showed downregulated expression of antioxidant enzymes including manganese superoxide dismutase (MnSOD), catalase (CAT), and glutathione peroxidase (GPx) (Fig. 1E–H); increased contents of malondialdehyde (MDA) (Fig. 1I); decreased cell activity (Fig. 1J); increased ROS levels (Fig. 1K, L); decreased mitochondrial membrane potential (MMP) (Fig. 1M, N); and increased rate of apoptosis (Fig. 1O, P). However, these results were reversed in PARK7-overexpressing BMSCs, which showed upregulated expression of MnSOD, CAT, and GPx (Fig. 1E–H); decreased intracellular ROS (Fig. 1K, L) and MDA levels (Fig. 1I); increased MMP expression (Fig. 1M, N) and cell activity (Fig. 1J); and decreased rate of apoptosis (Fig. 1O, P). Conversely, when PARK7 expression was downregulated in BMSCs, the results obtained using the abovementioned indicators were the opposite of those obtained using PARK7 overexpression (Fig. 1E–P). These findings suggest that PARK7 enhanced the ability of BMSCs to resist OS and promoted BMSC survival under conditions of OS.

### The Nrf2 signaling pathway participates in reversal of OS in BMSCs

**Fig. 2:**
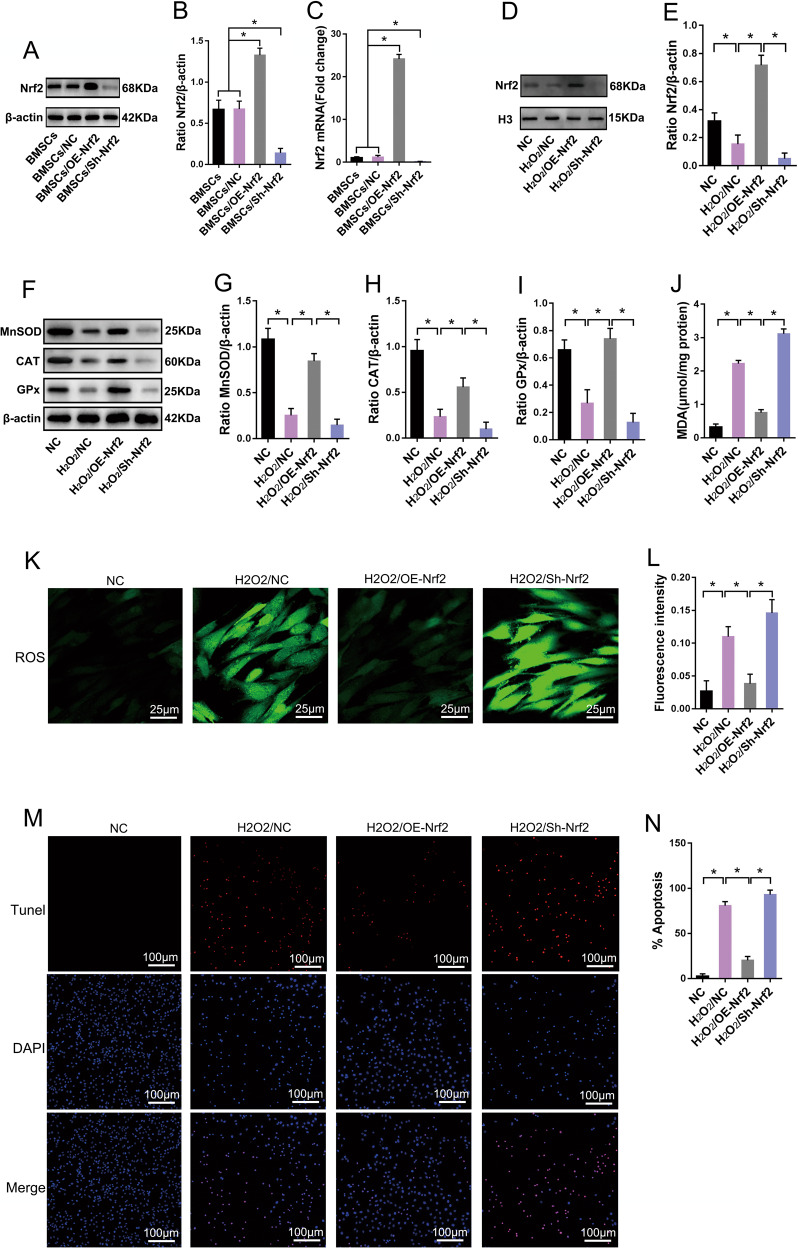
Nrf2 signaling pathway participates in reversing oxidative stress in BMSCs. After BMSCs were transfected with the Nrf2 overexpression or Nrf2 interfering lentivirus. **A** immunoblot analysis of Nrf2 protein expression in BMSCs (*n* = 3). *Nrf2* nuclear factor (erythroid-derived 2)-like 2, *Sh-Nrf2* short-hairpin ribonucleic acid of *Nrf2*, *OE-Nrf2* overexpression of Nrf2. **B** Quantification of Nrf2 expression is shown in **A** (*n* = 3). **C** RT-qPCR analysis of Nrf2 mRNA expression in BMSCs (*n* = 3). After Nrf2 was upregulated or downregulated in BMSCs, BMSCs were treated with H_2_O_2_ to simulate oxidative stress. **D** Immunoblot analysis of Nrf2 protein expression in BMSC nuclei (*n* = 3). H3, histone 3. **E** Quantification of Nrf2 expression is shown in **D** (*n* = 3). **F** Immunoblot analysis of MnSOD, CAT, and GPx protein expression in BMSCs (*n* = 3). **G** Quantification of MnSOD expression is shown in **F** (*n* = 3). **H** Quantification of CAT expression is shown in **F** (*n* = 3). **I** Quantification of GPx expression is shown in **F** (*n* = 3). **J** Detection of MDA levels using thiobarbituric acid assay (*n* = 6). **K** Detection of ROS levels using DCFH-DA (*n* = 5). *DCFH-DA* dichlorodihydrofluorescein diacetate. **L** Quantification of ROS levels is shown in **K** (*n* = 5). **M** Analysis of apoptosis activity using TUNEL/DAPI staining (*n* = 5). *TUNEL* terminal deoxynucleotidyl transferase deoxyuridine-5′-triphosphate nick end labeling. **N** Quantification of TUNEL-positive signal in BMSCs is shown in **M** (*n* = 5). All data are represented as mean ± SD. *P < 0.05. Differences were evaluated using one-way ANOVA with Tukey’s post hoc test (**B**–**C**, **E**, **G**–**J**, **L**, **N**).

The antioxidant enzymes MnSOD, CAT, and GPx, and downstream effector proteins of the nuclear factor (erythroid-derived 2)–like 2 (Nrf2) signaling pathway, are crucial for the elimination of intracellular ROS from cells [20, 21]. Therefore, we further studied the role of the Nrf2 signaling pathway in reducing OS in BMSCs. We transfected BMSCs with an Nrf2-overexpressing lentivirus (OE-Nrf2) or interference lentivirus (Sh-Nrf2) to upregulate or downregulate the expression of Nrf2 in BMSCs. The results of qPCR and immunoblotting confirmed that Nrf2 expression was successfully upregulated or downregulated in BMSCs (Fig. 2A–C). We then treated BMSCs with 1000 μM H_2_O_2_ for 24 h. Our results show that after prolonged exposure to OS, the expression of MnSOD, CAT, and GPx were downregulated (Fig. 2F–I), the levels of MDA (Fig. 2J) and ROS (Fig. 2K, L) were increased, and the apoptotic rate was significantly increased (Fig. 2M, N) in BMSCs. However, upregulating the expression of Nrf2 in BMSCs significantly increased the content of free Nrf2 in the nucleus (Fig. 2D, E); increased cellular expression of MnSOD, CAT, and GPx (Fig. 2F–I); decreased the levels of MDA and ROS (Fig. 2J–L); and significantly decreased the apoptotic rate (Fig. 2M, N) compared with the values obtained in the H_2_O_2_/NC group. Downregulation of Nrf2 expression yielded results that were opposite of those obtained using upregulation of Nrf2 expression (Fig. 2D–N). These findings suggest that the Nrf2 signaling pathway enhanced resistance to OS in BMSCs by regulating the expression of antioxidant enzymes, such as MnSOD, CAT, and GPx, which are crucial for maintaining cellular redox homeostasis.

### PARK7 protects BMSCs from OS by regulating the Nrf2 signaling pathway

**Fig. 3:**
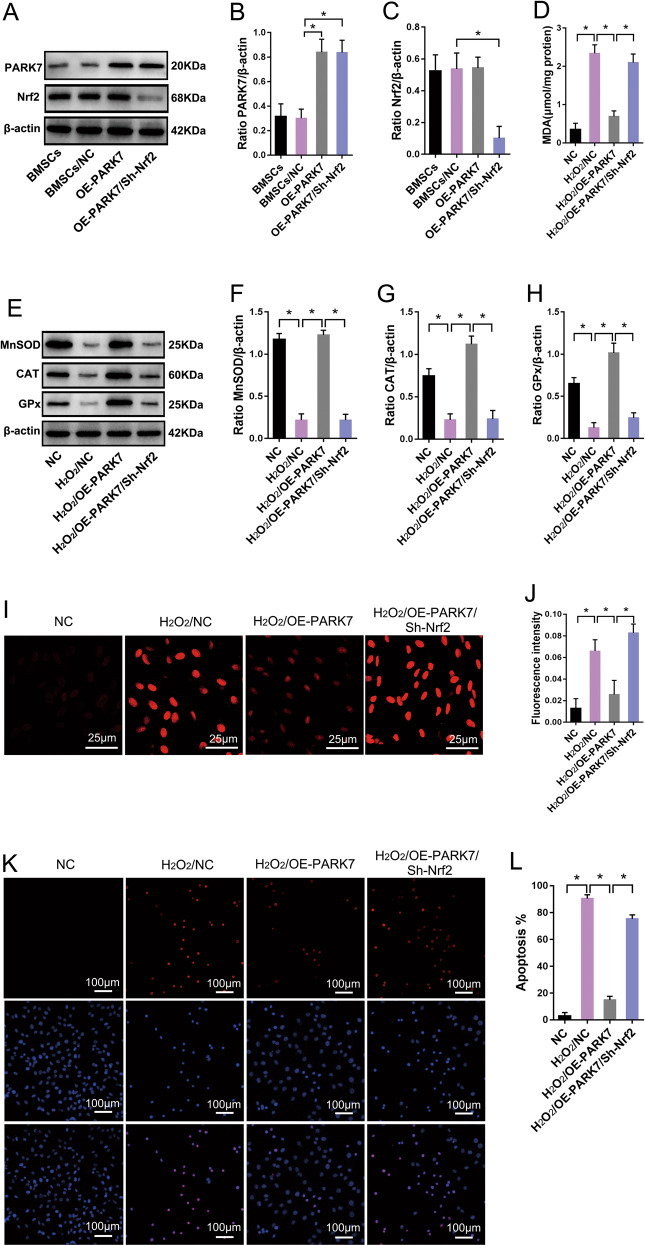
PARK7 protects BMSCs from oxidative stress by regulating the Nrf2 signaling pathway. After BMSCs were transfected with the PARK7 overexpression or Nrf2 interfering lentivirus. **A** Immunoblot analysis of PARK7 and Nrf2 protein expression in BMSCs (*n* = 5). **B** Quantification of PARK7 expression is shown in **A** (*n* = 5). **C** Quantification of Nrf2 expression is shown in **A** (*n* = 5). After PARK7 or Nrf2 expression was successfully up or downregulated in BMSCs, BMSCs were treated with H_2_O_2_ to simulate oxidative stress. **D** Detection of MDA levels using thiobarbituric acid (*n* = 6). **E** Immunoblot analysis of MnSOD, CAT, and GPx protein expression in BMSCs (*n* = 3). **F** Quantification of MnSOD expression is shown in **E** (*n* = 3). **G** Quantification of CAT expression is shown in **E** (*n* = 3). **H** Quantification of GPx expression is shown in **E** (*n* = 3). **I** Detection of ROS levels using DHE (*n* = 5). **J** Quantification of ROS levels is shown in **I** (*n* = 5). **K** Analysis of apoptosis levels using TUNEL/DAPI staining (*n* = 5). **L** Quantification of TUNEL-positive signal in BMSCs is shown in **K** (*n* = 5). All data are represented as mean ± SD. *P < 0.05. Differences were tested using one-way ANOVA with Tukey’s post hoc test (**B**–**D**, **F**–**H**, **J**, **L**).

Our results suggest that by regulating the expression of antioxidant enzymes, such as MnSOD, CAT, and GPx, the Nrf2 signaling pathway can enhance resistance to OS in BMSCs (Fig. 2). Functional experiments involving PARK7 show that PARK7 can also promote the expression of these antioxidant enzymes and enhance the ability to resist OS in BMSCs (Fig. 1). Therefore, we hypothesized that PARK7 can protect BMSCs from OS by regulating the Nrf2 signaling pathway. To verify this hypothesis, we first transfected BMSCs with PARK7-overexpressing lentivirus to upregulate the expression of *PARK7* in BMSCs (Fig. 3A, B); then, we treated these BMSCs with 1000 μM H_2_O_2_ for 24 h. Our results show that BMSCs subjected to extended exposure to OS downregulated their expression of MnSOD, CAT, and GPx (Fig. 3E–H) and increased their levels of MDA and ROS (Fig. 3D, I, J); in addition, numerous cells underwent stress-induced apoptosis (Fig. 3K, L). However, in BMSCs-overexpressing PARK7 and subjected to extended exposure to OS, the expression of MnSOD, CAT, and GPx was upregulated (Fig. 3E–H), whereas MDA and ROS levels were decreased (Fig. 3D, I, J), which can effectively inhibit OS-induced apoptosis (Fig. 3K, L). Based on the results obtained using upregulation of PARK7 expression, we then downregulated the expression of Nrf2 in BMSCs (Fig. 3A, C) and treated these BMSCs with 1000 μM H_2_O_2_ for 24 h. In these cells, the expression of MnSOD, CAT, and GPx was downregulated (Fig. 3E–H), whereas MDA and ROS levels, and rate of apoptosis, were increased (Fig. 3D, I–L). These findings show that downregulation of Nrf2 expression weakened PARK7-mediated protection against OS in BMSCs. Taken together, these results confirm that PARK7 protected BMSCs from OS-induced stress by regulating the Nrf2-signaling pathway.

### PARK7 regulates the Nrf2 signaling pathway by promoting dissociation of the Keap1–Nrf2 complex

**Fig. 4:**
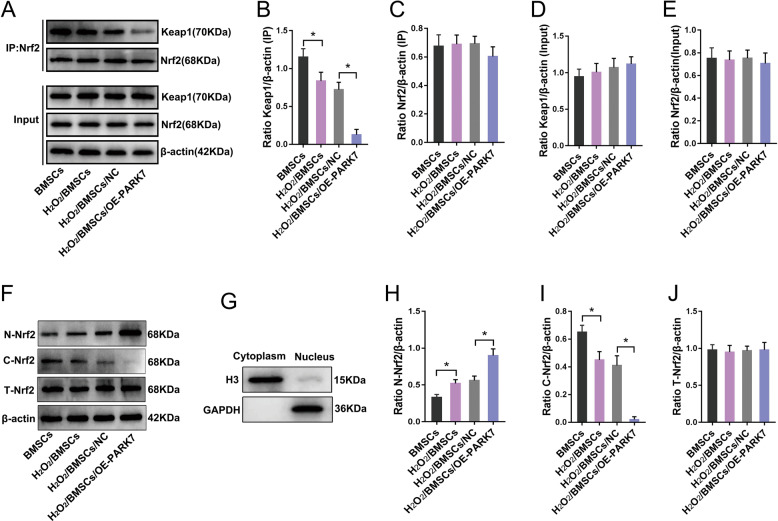
PARK7 regulates the Nrf2 signaling pathway. The effect of PARK7 on the interaction between Nrf2 and Keap1. **A** Immunoprecipitation and immunoblot analysis of Nrf2-Keap1 interactions in BMSCs (*n* = 3). *Keap1* Kelch-like echinacoside (ECH)–associated protein 1. **B** Quantification of immunoprecipitated Keap1 is shown in **A** (*n* = 3). **C** Quantification of immunoprecipitated Nrf2 is shown in **A** (*n* = 3). **D** Quantification of input Keap1 is shown in **A** (*n* = 3). **E** Quantification of input Nrf2 is shown in **A** (*n* = 3). PARK7 affects the entry of free Nrf2 into the nucleus. **F** Immunoblot analysis of N-Nrf2, C-Nrf2, and T-Nrf2 protein expression in BMSCs (*n* = 3). *N-Nrf2* nuclear Nrf2, *C-Nrf2* cytoplasmic Nrf2, *T-Nrf2* total intracellular Nrf2. **G** Immunoblot analysis of the purity of cytosolic and nuclear proteins (*n* = 3). Histone 3 (H3) was used as reference nuclear protein and glyceraldehyde-3-phosphate dehydrogenase (GAPDH) as reference cytoplasmic protein. **H** Quantification of N-Nrf2 expression is shown in **F** (*n* = 3). **I** Quantification of C-Nrf2 expression is shown in **F** (*n* = 3). **J** Quantification of T-Nrf2 expression is shown in **F** (*n* = 3). All data are represented as mean ± SD. *P < 0.05. Differences were tested using one-way ANOVA with Tukey’s post hoc test (**B**–**E**, **H**–**J**).

Under conditions of OS, the Nrf2 transcription factor binds to *Kelch*-like echinacoside (ECH)–associated protein 1 (Keap1) in the cytoplasm to form the Keap1–Nrf2 complex [22]. When stimulated by OS, Nrf2 dissociates from Keap1. Then, the free Nrf2 translocates to the nucleus and binds to antioxidant-response elements with the assistance of the musculoaponeurotic fibrosarcoma oncogene homolog protein, thereby initiating the transcription and expression of downstream genes encoding antioxidant enzymes [23, 24]. To investigate the mechanism underlying PARK7-mediated regulation of the Nrf2 signaling pathway, we first treated PARK7-overexpressing BMSCs with H_2_O_2_ to simulate conditions of OS. Then, we used immunoprecipitation with an antibody specific for Nrf2 to analyze the effect of PARK7 expression on the dissociation of the Keap1–Nrf2 complex. Our results show that under conditions of OS, PARK7 did not regulate the content of total Nrf2 and Keap1 in BMSCs (Fig. 4A, D, E), but did affect the interaction between Nrf2 and Keap1 (Fig. 4A–C). Partial dissociation occurred in both H_2_O_2_/BMSC and H_2_O_2_/BMSC/NC groups (Fig. 4A–C). In contrast, the dissociation of Keap1–Nrf2 complex increased in the group overexpressing PARK7 (Fig. 4A–C). Having established that PARK7 promoted the dissociation of the Keap1–Nrf2 complex, we next investigated whether PARK7 could promote the entry of free Nrf2 into the nucleus for enhanced transcriptional activation. For this, we isolated the Nrf2 protein from the nucleus and cytoplasm (Fig. 4G). Our results show that under conditions of OS, a scant amount of Nrf2 entered the nucleus in H_2_O_2_/BMSC and H_2_O_2_/BMSC/NC groups (Fig. 4F, H), whereas the levels of cytoplasmic Nrf2 were slightly reduced (Fig. 4F, I). In contrast, the level of free Nrf2 in the nucleus was significantly increased in the PARK7 overexpression group (Fig. 4F, H), the content of cytoplasmic Nrf2 was significantly reduced (Fig. 4F, I), whereas the total Nrf2 content in each group remained unchanged (Fig. 4F, J). These results indicate that PARK7 regulated the Nrf2 signaling pathway by promoting dissociation of the Keap1–Nrf2 complex and entry of free Nrf2 into the nucleus.

### PARK7 promotes BMSC survival in the OS microenvironment of the femoral head necrotic area

**Fig. 5:**
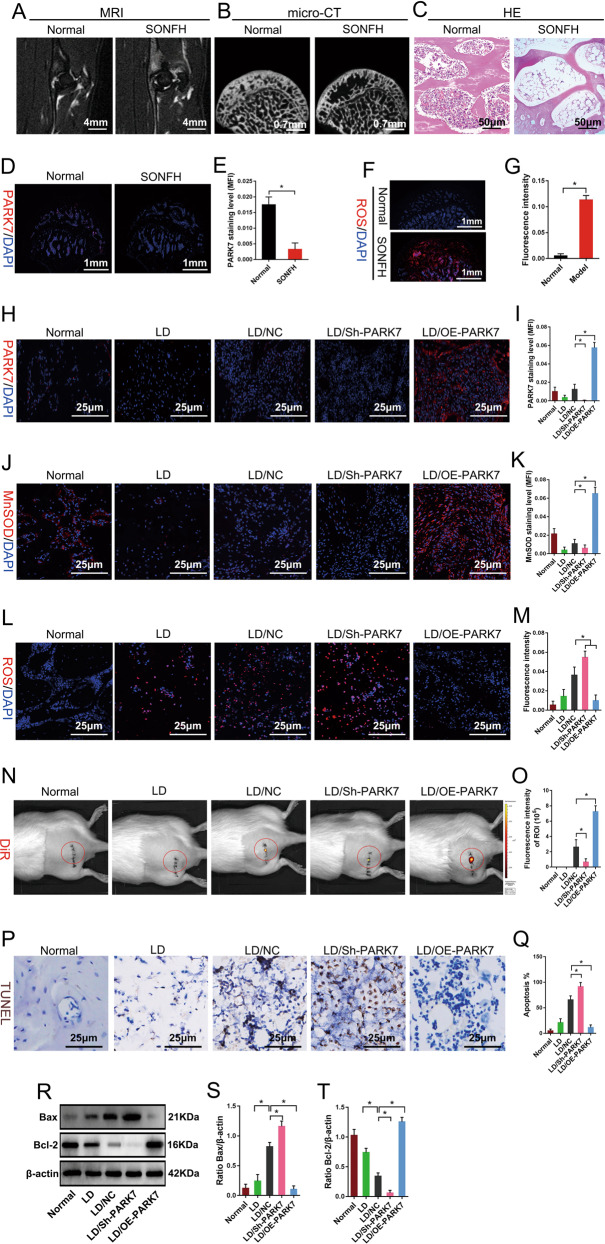
PARK7 promotes BMSC survival in the oxidative stress microenvironment of the femoral head necrotic area. At 6 weeks after the establishment of the early-SONFH model using treatment with methylprednisolone. **A** MRI assessment of osteonecrosis (*n* = 6). **B** Micro-CT assessment of osteonecrosis (*n* = 6). **C** HE staining of osteonecrotic areas (*n* = 6). **D** Expression level of PARK7 in the necrotic area of femoral head was detected by immunofluorescence labeling (*n* = 6). **E** Quantification of PARK7 expression levels is shown in **D** (*n* = 6). **F** The level of ROS in the necrotic area of femoral head was detected by DHE staining (*n* = 6). **G** Quantification of ROS levels is shown in **F** (*n* = 6). At 48 h after BMSC transplantation, **H** Expression level of PARK7 in the transplanted area was detected using immunofluorescence labeling (*n* = 6). *LD* lesion debridement. **I** Quantification of PARK7 expression levels are shown in **H** (*n* = 6). **J** Expression level of MnSOD in the transplanted area was detected using immunofluorescence labeling (*n* = 6). **K** Quantification of MnSOD expression levels is shown in **J** (*n* = 6). **L** Level of ROS in the transplanted area was detected using DHE staining (*n* = 6). **M** Quantification of ROS levels is shown in **L** (*n* = 6). **N** Live-animal imaging shows DiR fluorescence intensity in the transplanted area (*n* = 6). **O** Quantification of DiR fluorescence intensity is shown in **N** (*n* = 6). **P** Detection of BMSC apoptosis in the transplanted area using TUNEL staining (*n* = 6). **Q** Quantification of TUNEL-positive signal in BMSCs is shown in **P** (*n* = 6). **R** Immunoblot analysis of Bax and Bcl-2 protein expression in the transplanted area (*n* = 5). *Bcl-2* B-cell lymphoma 2, *Bax* Bcl-2-associated X protein. **S** Quantification of Bax expression is shown in **R** (*n* = 5). **T** Quantification of Bcl-2 expression is shown in **R** (*n* = 5). All data are represented as mean ± SD. *P < 0.05. A two-tailed unpaired Student’s *t* test was used for comparative analyses involving two groups (**E**, **G**). One-way ANOVA with Tukey’s post hoc test was used for analyses involving more than two groups (**I**, **K**, **M**, **O**, **Q**, **S**, **T**).

We used methylprednisolone to establish a rat model of early-stage SONFH. At 6 weeks after induction of SONFH, magnetic resonance imaging (MRI; T2-weighted image [WI]) showed mixed signals of different heights in the femoral head (Fig. 5A); micro-computed tomography (micro-CT) showed that absorption of subchondral trabecular bone in the weight-bearing area of femoral head increased, with areas of trabecular bone becoming thinner and sparser or even disappeared entirely (Fig. 5B); HE staining showed that medullary cavities contained a large amount of adipose tissue, and that numerous empty lacunae were present in trabecular bone (Fig. 5C). These results show that the rat model of early-stage SONFH was established successfully. Our previous in vitro results showed that glucocorticoids could inhibit the expression of PARK7 in BMSC (Fig. S2). Subsequently, we evaluated PARK7 expression and ROS levels in early steroid-induced femoral head necrosis. The results showed that compared with normal femoral heads, the expression of PARK7 was downregulated (Fig. 5D, E), the level of ROS was significantly higher in femoral head necrosis, and an OS microenvironment had formed in the area of femoral head necrosis (Fig. 5F, G). Finally, we investigated whether PARK7 could promote the survival of BMSCs in the femoral head necrotic area. For this, we used 1,1-dioctadecyl-3,3,3,3-tetramethylindotricarbocyanine iodide (DiR) to label BMSCs that overexpressed or underexpressed PARK7, and then transplanted these BMSCs into the early-stage SONFH rats. At 48 h post surgery, we assessed the effect of PARK7 on OS using MnSOD and ROS levels in transplantation sites. We then assessed the effect of PARK7 on BMSC apoptosis using DiR fluorescence, terminal deoxynucleotidyl transferase dUTP nick end labeling (TUNEL), and expression of B-cell lymphoma 2 (Bcl-2) and Bcl-2–associated X protein (Bax) in transplantation sites. Our results show PARK7 expression in transplantation sites of the lesion debridement (LD)/BMSC/Sh-PARK7 group was downregulated compared with that of the LD/BMSC/NC group (Fig. 5H, I). Downregulation of PARK7 expression decreased the expression of MnSOD (Fig. 5J, K), increased the level of ROS (Fig. 5L, M), decreased the fluorescence intensity of DiR (Fig. 5N, O), increased the proportion of TUNEL^+^ cells (Fig. 5P, Q), downregulated the expression of Bcl-2, and upregulated that of Bax in transplantation sites (Fig. 5R–T). However, upregulation of PARK7 expression effectively reversed the above results while reducing the rate of BMSC apoptosis and promoting their survival in transplantation sites under conditions of OS (Fig. 5H–T). These results suggest that PARK7 inhibited stress-induced apoptosis and promoted the survival of BMSCs transplanted into the necrotic area of the femoral head.

### PARK7 assists in repair of early-stage SONFH by promoting resistance to stress-induced apoptosis in BMSCs

**Fig. 6:**
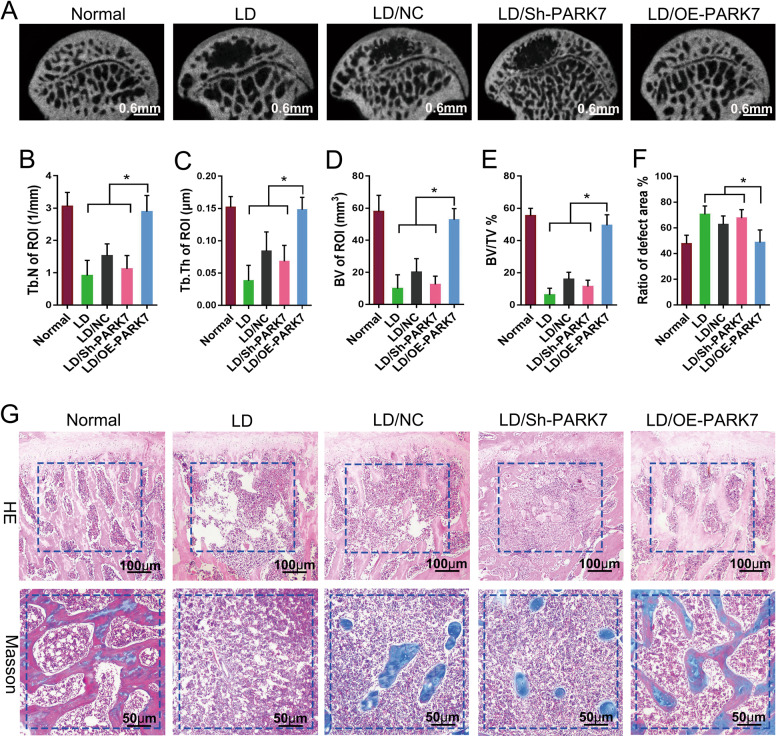
PARK7 promotes repair in early-stage SONFH by enhancing resistance to stress-induced apoptosis in BMSCs. At 12 weeks after BMSC transplantation. **A** Representative micro-CT images showing osteogenesis in the transplanted area (*n* = 6). **B** Quantification of Tb.N in the transplanted area is shown in **A** (*n* = 6). *Tb.N* number of trabeculae. **C** Quantification of Tb.Th in the transplanted area is shown in **A** (*n* = 6). *Tb.Th* trabecular thickness. **D** Quantification of BV in the transplanted area is shown in **A** (*n* = 6). *BV* bone volume. **E** Quantification of BV/TV in the transplanted area is shown in **A** (*n* = 6). *TV* tissue volume. **F** Quantification of defect area is shown in **A** (*n* = 6). **G** HE and Masson trichrome staining were used to evaluate the repair of bone defects (*n* = 6). All data are represented as mean ± SD. *P < 0.05. Differences were tested using one-way ANOVA with Tukey’s post hoc test (**B**–**F**).

Next, we investigated whether PARK7 could stimulate repair in early-stage SONFH by promoting resistance to stress-induced apoptosis in BMSCs. At 12 weeks after BMSC transplantation, we evaluated repair in the bone defect area using micro-CT, and HE and Masson trichrome staining. Micro-CT showed weak repair activity around the defect area in the LD, LD/BMSC/NC, and LD/BMSC/Sh-PARK7 groups, and the defect area was not completely repaired (Fig. 6A). The bone defect area in the LD/BMSC/OE-PARK7 group was completely repaired compared with that in the LD/BMSC/NC group (Fig. 6A). The LD/BMSC/OE-PARK7 group also showed significant increases in the trabecular number (Tb.N; Fig. 6B), trabecular thickness (Tb.Th; Fig. 6C), new bone volume (BV; Fig. 6D), and BV fraction (Fig. 6E), and the size of defect area was significantly decreased (Fig. 6F). These results were statistically significant (*P* < 0.05) when compared with the LD/BMSC/NC group but were not statistically significant when compared with the healthy control group. HE and Masson trichrome staining showed that a scant amount of new bone tissue was formed in the defect area of the LD, LD/BMSC/NC, and LD/BMSC/Sh-PARK7 groups (Fig. 6G). In contrast, a considerable amount of new bone tissue was formed in the defect area of the LD/BMSC/OE-PARK7 group. In addition, this new bone tissue tended to mature and resembled that in the normal femoral head (Fig. 6G). These findings suggest that PARK7 can impart resistance to stress-induced apoptosis in BMSCs, thereby improving the therapeutic effectiveness of BMSCs in early-stage SONFH.

## Discussion

The incidence of SONFH has been increasing yearly, and currently, SONFH ranks first among conditions involving nontraumatic necrosis of the femoral head [25]. If femoral head necrosis is not treated in time, the femoral head collapse rate within 2 years is >80%, and the resulting disability rate is high, highlighting the importance of early treatment [26]. BMSC transplantation is a new method for the repair of early-stage SONFH. However, a large number of transplanted BMSCs undergo apoptosis due to OS in the necrotic area of the femoral head, which greatly limits transplantation efficacy [5, 27, 28]. Inhibiting stress-induced apoptosis of BMSCs is key to resolving this issue. In this study, we investigated the role of PARK7 in promoting resistance to stress-induced apoptosis in BMSCs. Our results show that PARK7 promoted disintegration of the Keap1–Nrf2 complex, which led to activation of Nrf2. The activated Nrf2 entered the nucleus and initiated the expression of antioxidant enzymes such as MnSOD, CAT, and GPx, which eliminate excessive cellular ROS and protect cells from OS-induced injury and stress-induced apoptosis. We then investigated whether PARK7-mediated resistance to stress-induced apoptosis could improve the transplantation efficacy of BMSCs in early-stage SONFH. For this, we transplanted BMSCs-overexpressing PARK7 into rats with induced early-stage SONFH. Our results indicate that PARK7 overexpression effectively imparted resistance to stress-induced apoptosis in BMSCs transplanted into the osteonecrotic area, thereby improving the transplantation efficacy of BMSCs in early-stage SONFH.

OS is an important pathophysiological mechanism in SONFH [6, 7]. Dysfunction of the cellular mitochondrial aerobic respiratory chain results in the production of excessive ROS due to hypoxia in the femoral head necrotic area [29]. After the femoral head becomes necrotic, numerous inflammatory cells infiltrate, and these inflammatory cells will also produce excessive ROS in BMSCs [30]. Excessive ROS can change the redox state of transplanted BMSCs and damage mitochondria, DNA, proteins, lipids, and other biomacromolecules, resulting in stress-induced BMSC apoptosis [31, 32]. Our results indicate that under conditions of OS, BMSCs decreased their expression of MMPs and increased those of ROS and MDA; consequently, numerous BMSCs underwent stress-induced apoptosis. Therefore, enhancing the ability of BMSCs to resist OS is conducive to their survival in the OS microenvironment, and will improve the transplantation efficacy of BMSCs in early-stage SONFH.

PARK7 is an antioxidative protein that can enhance cellular resistance against OS and stress-induced apoptosis [33, 34]. In our present study, the results of our functional experiments with PARK7 showed that PARK7 overexpression reduced ROS levels in BMSCs; increased the expression of MnSOD, CAT, and GPx; protected the mitochondria; and enhanced BMSC resistance to OS and stress-induced apoptosis. Conversely, downregulation of PARK7 expression led to a decrease in BMSC resistance to OS, and numerous BMSCs underwent stress-induced apoptosis under conditions of OS. Studies on neurons and cardiomyocytes have shown that PARK7 can clear excessive ROS and maintain mitochondrial function via auto-oxidation of cysteine residues at sequences 46, 53, and 106. PARK7 can also auto-oxidize to monitor OS and can activate extracellular signal-regulated kinase 1/2 (ERK1/2), apoptosis signal-regulating kinase 1 (ASK1), and phosphatidylinositide 3-kinases (PI3K)/Akt signaling pathways, thereby enhancing resistance to OS and inhibiting stress-induced apoptosis [35–40]. In this study, we investigated the role of the Nrf2 signaling pathway in PARK7-mediated resistance to stress-induced BMSC apoptosis. Our results indicate that PARK7 promoted the dissociation of Keap1–Nrf2 complex, thereby activating Nrf2. The activated Nrf2 then entered the nucleus to initiate the expression of *MnSOD*, *CAT*, *GPx*, and other genes encoding antioxidant enzymes. This cascade resulted in the removal of excessive cellular ROS and protected the cells from OS-induced injury and stress-induced apoptosis.

Our previous attempts to use BMSC transplantation in a model of early-stage SONFH resulted in unsatisfactory efficacy due to numerous BMSCs undergoing stress-induced apoptosis in the femoral head necrotic area [5]. Our results obtained in vitro confirmed that PARK7 effectively improved BMSC resistance to OS, enabling BMSCs to resist stress-induced apoptosis. To evaluate the effect of PARK7 in vivo, we transplanted BMSCs-overexpressing PARK7 into rats with early-stage SONFH. Our results show that PARK7 imparted resistance to stress-induced apoptosis in BMSCs transplanted into the osteonecrotic area, thereby improving the therapeutic efficacy of transplantation.

In conclusion, our study shows that PARK7 enhanced resistance to OS and inhibited stress-induced apoptosis in BMSCs by regulating the Nrf2 signaling pathway. This enhanced protection against OS improved the therapeutic efficacy of BMSCs used to repair early-stage SONFH. Our study provides a new approach for preventing stress-induced BMSC apoptosis and improves their therapeutic efficacy in early-stage SONFH.

## Materials and methods

### Animals

All animal studies were approved by the Experimental Animal Bioethics Committee of the Guizhou Medical University (GMU), Guiyang, China. All procedures involving animals conformed to the *Guide for the Care and Use of Laboratory Animals* in accordance with Directive 2010/63/EU of the European Parliament. BMSCs were extracted from young male Sprague Dawley (SD) rats (20–30 g, 60 rats). The model of SONFH was established in adult male SD rats (400–500 g, 150 rats). All SD rats were provided by the Laboratory Animal Center of GMU (no. 1800815).

### BMSC isolation and culture

Bilateral femurs and tibias were harvested under aseptic conditions from young male SD rats weighing 20–30 g. The medullary cavity was flushed with l-glutamine Dulbecco’s modified Eagle medium (l-DMEM; GIBCO [Thermo Fisher Scientific, Waltham, MA, USA). Bone marrow tissue was centrifuged at 1000 rpm for 5 min to remove suspended adipose tissue. Bone marrow precipitates were then resuspended in complete l-DMEM (10% fetal bovine serum (FBS) and 1% double-antibody (aB); GIBCO) and cultured at 37 °C and 5% CO_2_. When primary BMSCs reached 90% confluence, they were digested using 0.25% trypsin–0.02% ethylenediaminetetraacetic acid (EDTA; GIBCO) and passaged at a ratio of 1:3. Third-generation BMSCs were used for subsequent experiments.

### Identification of BMSC surface antigens using flow cytometry

First, 5 μL of mouse anti-CD90-PECyTM7, mouse anti-CD106-PE, mouse anti-CD11b-V450, and mouse anti-CD45-fluorescein isothiocyanate (FITC) (BD Biosciences, Franklin Lakes, NJ, USA) was added into an flow cytometry (FCM) tube, followed by the addition of 50 μL cell suspension (2 × 10^7^/mL). The mixture was incubated at room temperature (RT) in the dark for 30 min, and the contents of each tube were then washed twice using a standing buffer. Then, 500 μL staining buffer was added to resuspend the cells, and FCM (Beckman Coulter Life Sciences, Brea, CA, USA) was used to detect expression levels of CD11b, CD45, CD90, and CD106.

### BMSC identification using osteogenic, cartilage, and adipogenic differentiation

Third-generation BMSCs at 60–70% confluence were differentiated using osteogenic- or chondrogenic-differentiation medium (Cyagen Biosciences, Santa Clara, CA, USA). After 2 weeks of osteogenic induction, 0.1% Alizarin Red stain (Cyagen) was used to identify calcium nodules, and a modified Gomori calcium cobalt stain (Cyagen) was used to detect ALP activity. After 4 weeks of induction, an Alisin Blue stain (Cyagen) was used to identify acid mucopolysaccharides in cartilage. When the degree of cellular fusion of third-generation BMSCs reached 100%, an adipogenic-induction medium (Cyagen) was used to induce BMSC differentiation. After 3 weeks of induction, Oil Red O stain (Cyagen) we used to identify lipid droplets in the cells.

### BMSC model of OS

Third-generation BMSCs were treated using 1000 μM H_2_O_2_ for 24 h to mimic conditions of OS as described previously [5, 41].

### Lentiviral transfection and screening of mixed clones

PARK7 and Nrf2 overexpression and interference lentiviruses were purchased from China Shanghai Genechem Co., Ltd. (Shanghai, China). Based on pre-transfection experiments conducted to determine the optimal multiplicity of infection (100) and transfection conditions (HitransG P; China Shanghai Genechem), BMSCs were infected with the lentiviruses; NC and blank controls were processed concurrently using identical conditions. After a 10-h incubation, the medium was replaced with complete L-DMEM. On day 4 post-infection, 2 μg/mL purinomycin (China Shanghai Genechem) was used to screen the cells successfully transfected by lentivirus. After all the blank control cells died, the concentration of purinomycin was reduced to 1 μg/mL to maintain screening in order to obtain mixed clone cell lines.

### Real-time qPCR

RNA was extracted from BMSCs using column affinity purification (QIAGEN, Hilden, Germany). Complementary DNA was synthesized using M-MuLV RT Master Mix with Oligo(dT) (Sangon Biotech, Shanghai, China). RT-qPCR was performed on a StepOnePlus system (Applied Biosystems, Foster City, CA, USA) in 96-well plates using specific primers and SYBR Green Mix (Sangon Biotech). Rat primer (Sangon Biotech) sequences were as follows: *PARK7*-F: AGGCGAGC TGGGATTAAGGT; *PARK7*-R: GACGACCACATCATACGGGC; *Nrf2*-F: TTGTAGATGACCATGAGTC GC; *Nrf2*-R:ACTTCCAGGGGCACTGTCTA; *β-actin* (*ACTB*)- F: CACCCGCGAGT ACAACCTTC; *ACTB*-R: CCCATACCCACCATCACACC. Fold change values in RNA expression over that of control were calculated using the ^ΔΔ^Ct method.

### Immunoblotting

Cells were lysed using radioimmunoprecipitation assay cell lysate buffer (Beyotime Institute of Biotechnology, Shanghai, China). The lysates were centrifuged at 13,000 × *g*/min for 10 min, and protein concentration in the supernatants was determined using a bicinchoninic acid protein concentration detection kit (Solarbio, Beijing, China). Proteins were separated using sodium dodecyl sulfate–polyacrylamide gel electrophoresis and transferred to a polyvinylidene fluoride membrane (MilliporeSigma, Burlington, MA, USA). The membrane was first blocked in 5% bovine serum albumin at RT for 2 h and then incubated overnight at 4 °C with the following primary antibodies: rabbit anti-rat *PARK7* (1:5000; ab76008) and mouse anti-rat Nrf2 (1 µg/ml; ab89443; Abcam Cambridge, UK), rabbit anti-rat glyceraldehyde-3-phosphate dehydrogenase (1:5000; ab245357), rabbit anti-rat H3 (1 µg/ml; ab18521), mouse anti-rat Keap1 (1:1500; ab119403), rabbit anti-rat MnSOD (1:2000; ab68155), rabbit anti-rat CAT (1:1000; ab76110), rabbit anti-rat GPx-3 (1:1000; ab256470), and mouse anti-rat β-actin (1:2500; ab8226; Abcam). The membrane was incubated at RT for 1–2 h with horseradish peroxide (HRP)-conjugated goat anti-rabbit or HRP-conjugated goat anti-mouse immunoglobulin G (IgG; 1:3000; 7076 S; Cell Signaling Technology, Danvers, MA, USA) used as secondary antibodies. Protein expression was visualized using an ECL kit (MerckMillipore). Images were acquired using a gel imaging system (Clinx Science Instruments, Ltd., Shanghai, China) and quantitatively analyzed using ImageJ software version 1.4.3.67 (National Institutes of Health [NIH], Bethesda, MD, USA).

### Assessment of ROS expression

BMSCs were washed using phosphate-buffered saline (PBS) and incubated with dichlorodihydrofluorescein diacetate (Sigma, Munich, Germany) or dihydroethidium (Beyotime) for 30 min at 37 °C. The emission of red fluorescence was assessed using a Cy3 channel on a confocal microscope (Zeiss, Germany).

### Cell viability assay

BMSCs were washed with PBS, and then Cell Counting Kit-8 (Solarbio, Beijing, China) solution was added to each well at 10 μL. After the cells were incubated for 3 h, absorbance (optical density) for each well was measured at 450 nm using a microplate reader (BioTek, VT, USA).

### Malondialdehyde assay

BMSCs were lysed using ultrasound to obtain cellular lysates. Then, 100 μL lysate was added to an MDA kit working solution per manufacturer’s instructions (Beyotime). The mixture was heated to 100 °C for 15 min, cooled in a water bath, and centrifuged at 1000 g/min for 10 min. Next, supernatants were aliquoted into a 96-well plate at 200 μL per well. Absorbance was measured at 523 nm using a microplate reader (Biotech).

### Assessment of mitochondrial membrane potential

A mitochondrial membrane potential kit (KeyGen BioTech, Nanjing, China) was used to assess mitochondrial membrane potential (MMP) in BMSCs. In brief, BMSCs were washed with PBS, and a JC-1 reaction mixture was prepared per kit instructions. The reaction mixture was added to the cells and allowed to incubate at 37 °C for 30 min. Next, cells were washed three times with PBS, and fluorescence was evaluated using a laser confocal microscope (Carl Zeiss AG, Oberkochen, Germany).

### Apoptosis

#### TUNEL/DAPI

BMSCs were washed with PBS, fixed with 4% paraformaldehyde for 30 min at 25–30 °C, made transparent, and incubated with TUNEL detection solution (Beyotime, China) at 37 °C for 90 min. Next, the cells were incubated with 10 μg/mL 4′,6-diamidino-2-phenylindole (DAPI, Solarbio) at RT for 8 min, and then washed five times using PBS. Anti-fluorescence quenching sealing solution (Solarbio) was then used to seal the samples. Fluorescence was observed under a Zeiss laser confocal microscope (Carl Zeiss AG, Oberkochen, Germany).

#### Annexin V-FITC/PI

Cells were digested using 0.25% trypsin–0.02% EDTA (GIBCO), resuspended in 1× binding buffer (BD Biosciences), and cell concentration was adjusted to 5 × 10^6^/mL. Then, 100 μL cell suspension was transferred into a flow cytometry tube, and 5 μL Annexin V-FITC and 5 μL PI (both BD Biosciences) were added into the tube. The mixture was incubated at RT in the dark for 15 min. Finally, 400 μL 1× binding buffer was added to each tube, and apoptosis was analyzed using FCM (Beckman).

### Rat model of early-stage SONFH

First, 150 adult male SD rats weighing 400–500 × *g* received seven injections of methylprednisolone (60 mg/kg; Pfizer, Andover, MA, USA) via the gluteal muscles once per day. The rats were weighed before each injection. At week 6, we performed MRI and micro-CT examination on the animals and stained the femoral head tissue with hematoxylin and eosin. We then confirmed the success of early-SONFH modeling and used the model for in vivo experiments.

### Transplantation of BMSCs for the repair of early-stage SONFH in vivo

We used a random number table to divide 80 adult male SD rats with femoral head necrosis into four groups: LD, LD/BMSC/NC, LD/BMSC/Sh-PARK7, and LD/BMSC/OE-PARK7, 20 rats per group. We randomly selected 20 healthy adult male SD rats as healthy controls. The procedure was as follows. The rats received intramuscular injections of penicillin (50,000 units/kg; CSPC, Shijiazhuang, China) 1 h before surgery to prevent infection. Intraperitoneal injection of chloral hydrate (10%, 3 mL/Kg) was used to induce anesthesia. Fur was shaved from each rat’s left hip, and the rat was placed on a clean operating table in a prone position. The left hip was then disinfected with 2% iodophor and covered with a towel, which is a disposable, sterile, and non-woven surgical fabric. A straight incision ~3–4 cm in length was made at the center of 0.3–0.5 cm behind the left greater trochanter, perpendicular to the spine. The gluteus maximus muscle was bluntly separated to expose the anterior end of the gluteus medius muscle and the sciatic nerve. The sciatic nerve was pulled to the caudal side with a retractor, the gluteus medius muscle was pulled to the cephalic side using another retractor, and the tendon on the surface of the femoral neck was exposed and excised. Using straight forceps, the proximal end of the femur was clamped and rotated to the cephalic side, and the ganglion capsule was removed to expose the femoral head. A sterile drill equipped with a bit 1–2 mm in diameter was used to drill holes from the posterolateral to the anteromedial side of the femoral head to a depth of ~3 mm. Then, necrotic bone tissue was removed using a curette. A total of 1 × 10^7^ BMSCs labeled with DiR, which is used to trace BMSCs, were resuspended in 0.1-mL complete l-DMEM and injected into the femoral head via the tunnel that had been drilled into the bone. The drill holes were then sealed with bone wax, and the wound was closed. The rats were then placed into their cages for recovery, and penicillin (50,000 units/kg) was administered to the rats for 3 days post surgery to prevent infection.

### Live imaging of animals

BMSCs were labeled with DiR (Yeasen Biotechnology Co., Ltd., Shanghai, China) fluorescent dyes before surgery. At 48 h after BMSC transplantation, the rats were anesthetized using an intraperitoneal injection of chloral hydrate (10%, 3 mL/kg), placed on the imaging platform, and imaged using a small-animal imaging system (PerkinElmer, MA, USA). Fluorescence intensity in the femoral head necrotic area was then calculated using Living Image software (PerkinElmer).

### Micro-CT

At 12 weeks after BMSC transplantation, the femoral head tissues were harvested and fixed in paraformaldehyde (4%, 2 days). The tissues were then scanned using micro-CT with a high-resolution system to evaluate bone repair. In brief, the tissue samples were scanned continuously at a resolution of 6.5 µm per voxel. The defect area of the femoral head is the region of interest (ROI). Tb.N, Tb.Th, BV, BV fraction, and the size of the defect area were then calculated for each ROI. NRecon software (Micro Photonics Inc., Allentown, PA, USA) was used for 3D image reconstruction, and CTAn software (Bruker) was used for 3D analysis.

### HE and Masson trichrome staining

At 12 weeks after BMSC transplantation, the bone tissue was decalcified in 10% EDTA solution at 37 °C for 2 months, dehydrated, embedded in paraffin, and sectioned at the thickness of 3 μm. The sections were deparaffinized, rehydrated, and stained using an HE staining kit (Solarbio, China) or a Masson trichrome staining kit (Solarbio, China) per instructions of the respective manufacturer. The sections were then dehydrated, cleared in xylene, sealed with neutral balsam, and examined under a biological microscope (OLYMPUS BX53, Tokyo, Japan).

### Statistical analysis

All statistical data were analyzed and graphed using GraphPad Prism software version 6 (GraphPad Software, San Diego, CA, USA). The Kolmogorov–Smirnov test was used to analyze normally distributed data. A two-tailed unpaired Student’s *t* test was used for comparative analyses involving two groups. One-way analysis of variance with Tukey’s post hoc test was used for analyses involving more than two groups. All error bars are expressed as mean ± SD. *P* < 0.05 was considered statistically significant.

## Supplementary information

Supplementary figure 1

Supplementary figure 2

Supplementary figure legends

## Data Availability

Additional data or reagents are available from the corresponding author upon reasonable request.
