# Supplementary figures and images for: PARK7 promotes repair in early steroid-induced osteonecrosis of the femoral head by enhancing resistance to stress-induced apoptosis in bone marrow mesenchymal stem cells via regulation of the Nrf2 signaling pathway
Source: Cell Death Dis. 2021 Oct 13;12(10):940. doi: 10.1038/s41419-021-04226-1 (PMC8514492; doi:10.1038/s41419-021-04226-1)

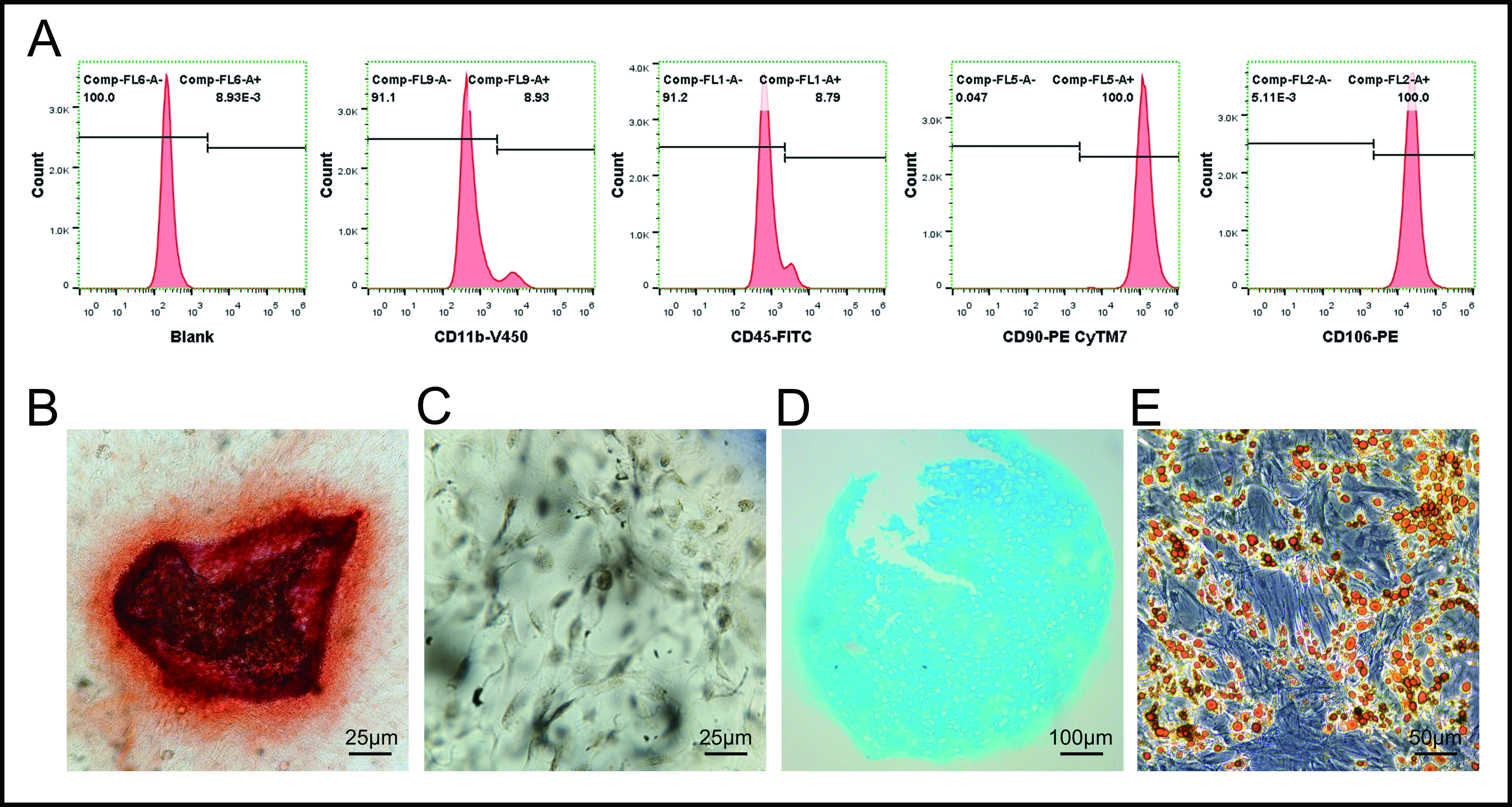

Supplement: Supplementary file 1 — Supplementary figure 1 [file 41419_2021_4226_MOESM1_ESM.jpg]

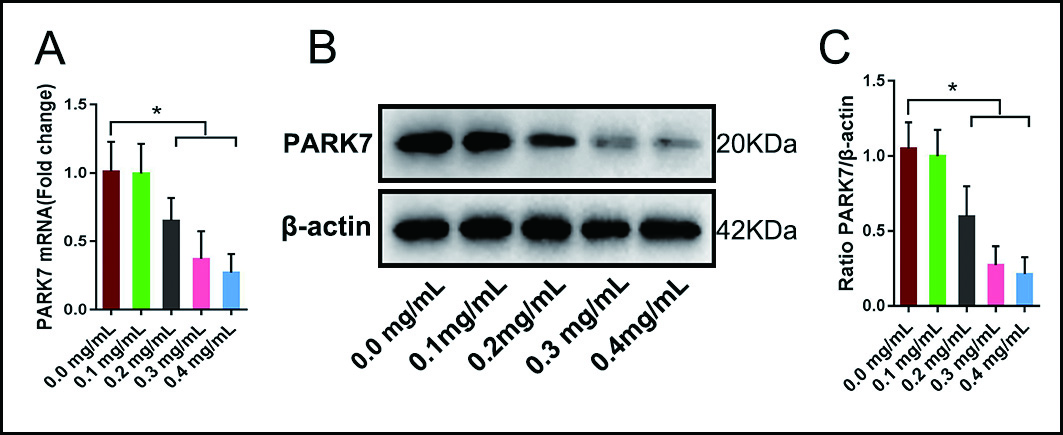

Supplement: Supplementary file 2 — Supplementary figure 2 [file 41419_2021_4226_MOESM2_ESM.jpg]
