# Supplementary material for: PARK7 promotes repair in early steroid-induced osteonecrosis of the femoral head by enhancing resistance to stress-induced apoptosis in bone marrow mesenchymal stem cells via regulation of the Nrf2 signaling pathway
Source: Cell Death Dis. 2021 Oct 13;12(10):940. doi: 10.1038/s41419-021-04226-1 (PMC8514492; doi:10.1038/s41419-021-04226-1)
Supplement: Supplementary file 3 — Supplementary figure legends [file 41419_2021_4226_MOESM3_ESM.doc]

**Supplementary figure legends:**

**Fig. S1 Isolation, culture, and identification of BMSCs:** **A** FCM analysis of BMSC surface antigens CD11b, CD45, CD90 and CD106 (n = 5). **B** Representative images showing Alizarin Red staining after 2 weeks of BMSC osteogenic induction (n = 5). **C**Representative images showing ALP staining after 2 weeks of BMSC osteogenic induction (n = 5). ALP, alkaline phosphatase. **D** Representative images showing Alisin Blue staining after 4 weeks of BMSC chondrogenic induction (n = 5). **E**Representative images showing Oil Red O staining after 3 weeks of BMSC adipogenic induction (n = 5).

**Fig. S2 The effect of glucocorticoids on the expression of PARK7 in BMSC:** **A** RT-qPCR analysis of PARK7 mRNA expression in BMSCs (n = 3). **B** Immunoblot analysis of PARK7 protein expression in BMSCs (n = 3). **C** Quantification of PARK7 expression is shown in **B** (n = 3). All data are represented as mean ± standard deviation (SD). ^*^*P* < 0.05. Differences were assessed using a one-way analysis of variance (ANOVA) with Tukey’s *post hoc* test (**A**, **C**).
